# Update on parasitic dermatoses^☆^^☆☆^

**DOI:** 10.1016/j.abd.2019.12.001

**Published:** 2019-12-31

**Authors:** Alberto Eduardo Cox Cardoso, Alberto Eduardo Oiticica Cardoso, Carolina Talhari, Monica Santos

**Affiliations:** a Professor Alberto Antunes University Hospital, Universidade Federal de Ciências da Saúde de Alagoas, Maceió, AL, Brazil; b Department of Dermatology, Universidade Estadual de Ciências da Saúde de Alagoas, Maceió, AL, Brazil; c Graduate Program of the Universidade do Estado do Amazonas, Manaus, AM, Brazil; d Department of Teaching and Research, Fundação Alfredo da Matta de Dermatologia, Manaus, AM, Brazil; e Department of Dermatology, Universidade do Estado do Amazonas, Tropical Dermatology Outpatient Clinic, Fundação Alfredo da Matta de Dermatologia, Manaus, AM, Brazil

**Keywords:** Drug therapy, Larva migrans, Lice infestations, Myiasis, Onchocerciasis, Scabies, Skin diseases, parasitic, Tungiasis

## Abstract

These are cutaneous diseases caused by insects, worms, protozoa, or coelenterates which may or may not have a parasitic life. In this review the main ethological agents, clinical aspects, laboratory exams, and treatments of these dermatological diseases will be studied.

## Introduction

Parasitic diseases are skin conditions caused by insects, worms, protozoa, or coelenterates that may or may not be parasitic. Given the relatively easy movement of people to different regions of the planet, knowledge of these diseases is becoming increasingly important.

This review will address the main clinical and therapeutic aspects of scabies, pediculosis, myiasis, tungiasis, larva migrans, Lyme disease, and onchocerciasis.

## Scabies

Scabies is a contagious disease. The etiological agent is a mite, *Sarcoptes scabiei var. hominis*. Recent molecular epidemiological studies have shown that scabies caused by *S. scabiei var. hominis* is exclusively human (it does not affect animals), and transmission occurs through personal contact, with no predilection for age, ethnicity, or gender. Transmission by fomite is rare.^1^, ^2^

Scabies epidemiology is cyclical, especially in developed countries. The interval between cycles ranges from ten to 15 years, approximately.

According to studies on dust samples from infected patients’ homes, under normal environmental conditions, *S. scabiei* can survive outside the host for 24–36 h.^1^, ^2^

### Mite biology and morphology

After mating, the male dies, and the female penetrates the epidermis, digging furrows to lay eggs and feces.

In four to six weeks, females can release 40–50 eggs. After hatching, the hexapod larvae leave the furrows. The number of adult mites in an infected individual is estimated at 12 parasites.

### Clinical manifestations

Pruritus is the main symptom of scabies, being more intense at night. In most cases, this symptom begins insidiously, progressively intensifying. It can affect almost the entire body; the face is rarely affected.

The characteristic lesion is linear, serpiginous, and raised, measuring a few millimeters; at one end, a papular-vesicular lesion may be observed, described by some authors as a “black spot.” These lesions are most often observed on the lateral surfaces of the fingers, palmar regions, hands, wrists, and feet.

Scalp scabies is not common in adults; however, it may accompany or resemble seborrheic dermatitis.

Left untreated, erythematous papular lesions appear in the armpits, breasts, penis, buttocks, interdigital spaces, waist, and feet.

**Figure 1:**
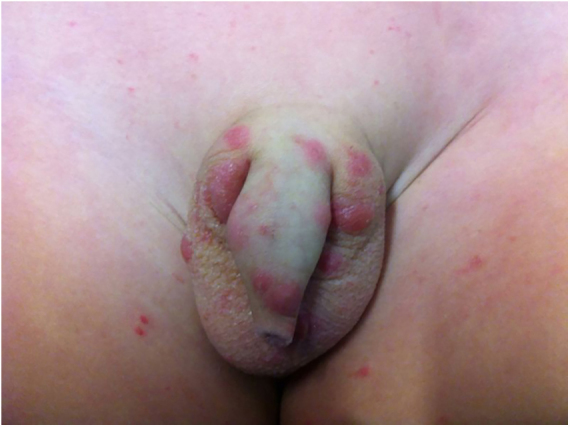
Nodular scabies – intense pruritus and erythematous papular nodular lesions.

Long-term patients may present papular-nodular reddish-brown lesions, mainly located on the genitalia, armpits, trunk, and elbows. These lesions are very itchy and their regression is slow, even after proper treatment. These manifestations are called nodular scabies (Fig. 1). It had been assumed that there was no mite in these lesions. However, recent studies have demonstrated the presence of *S. scabiei.*^3^, ^4^ In children, nodular scabies may simulate mastocytosis.^5^

In newborns and young children, the face and scalp may be affected, and cervical polymicroadenopathy may be observed. Eczema or hive lesions may hinder the diagnosis, especially in infants.^6^

In the elderly, the skin reaction to the presence of the mite may be less pronounced and cause atypical conditions. Dorsal injuries may be mistaken for senile pruritus. Vesicobullous lesions, which are clinically and histologically similar to bullous pemphigoid, have been reported in patients older than 6 years without debilitating disease.

**Figure 2:**
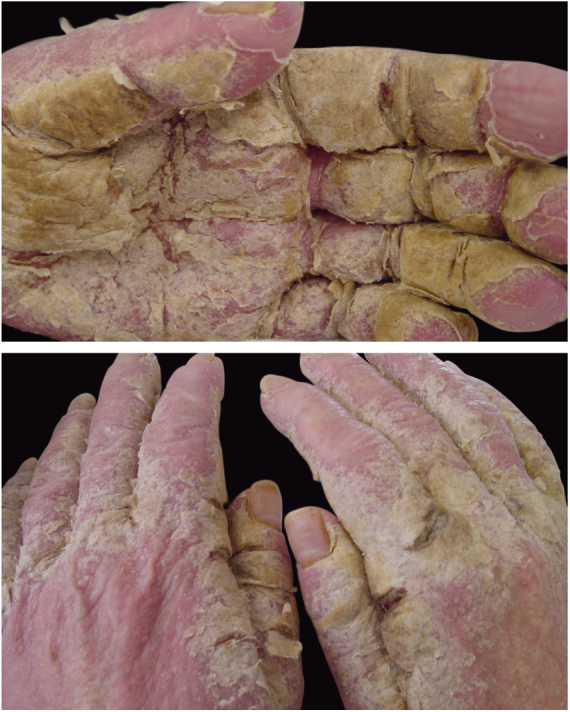
Crusted scabies. Intense, constant pruritus and generalized erythematous, squamous lesions. HTLV+ patient. Personl archive: Dr. Paulo Roberto Machado.

Crusted scabies, also known as Norwegian scabies, is a clinical variety of scabies that occurs due to mite hyperinfestation; over one million parasites can be found in this condition. Currently, it is observed primarily in immunosuppressed patients, those under chemoterapy, those with malignant neoplasms, and transplant recipients. The frequency of this clinical form has increased in HIV-positive patients.^7^, ^8^ Australian aborigines and carriers of the HTLV1 virus are also relatively frequently affected.^9^ Clinically, it is characterized by hyperkeratotic, crusted lesions with cutaneous fissures and thickened and dystrophic nails (Fig. 2). Secondary infections may be observed in these patients. In the vast majority of cases, the pruritus is very intense. The differential diagnosis is mainly made with Darier's disease and psoriasis.

### Differential diagnosis

Differential diagnosis is made with most pruritic conditions, such as atopic dermatitis, drug rash, papular urticaria, insect bites, and pyoderma.

### Diagnosis

Direct examination of the lesions should always be done, especially in atypical cases. Two drops of mineral oil are placed on the lesions, which are then scarified with a scalpel blade or curette, removing the furrow ceiling, which is placed on a glass slide. It is then examined under the microscope at 10× and 40× magnification. The mite, eggs, and/or feces may be observed. A few drops of 30% potassium hydroxide can be placed on the collected material prior to microscope observation.

PCR may be useful in clinically atypical cases.^10^

**Figure 3:**
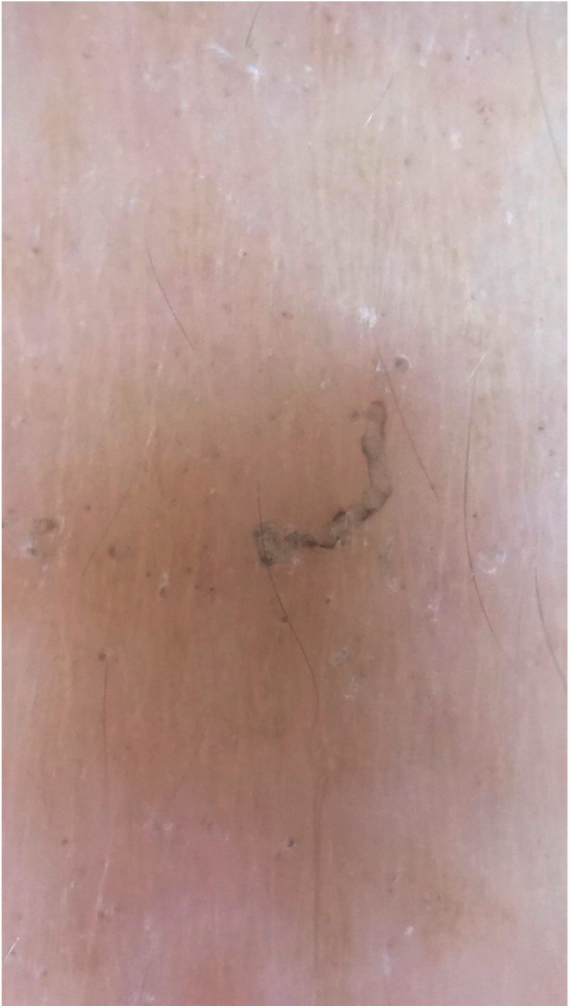
*Sarcoptes scabiei*. Dermoscopy. At the lower end, a “hang glider”-shaped dark spot, corresponding to the anterior segment of the mite.

Dermoscopy can also be used to find the parasite (Fig. 3).^11^ Epiluminescence microscopy is another recommended method for mite visualization;^12^ confocal microscopy and optical coherence tomography may also be used.^13^

### Treatment

In the treatment of scabies, it is important that all household residents are treated, in order to avoid reinfestation.

Permethrin: a synthetic, effective, and non-toxic pyrethroid is available as a 5% cream or lotion. It can be used in children, adults, pregnant women, and nursing mothers.

It should be applied over the entire body, from neck to toe. In children, it should also be applied to the scalp and retroauricular ridges. The drug should be applied at nighttime, for two consecutive nights. On the third day, in the morning, all bedding should be removed and washed.

Precipitated sulfur: recommended at concentrations of 5–10% in petroleum jelly. It does not cause side effects. It can be used in children, pregnant women, and nursing mothers. It should be applied on the entire body for four consecutive nights, and removed during the day.

Ivermectin: used systemically. It is a semi-synthetic macrocyclic lactone suitable for adults and children over 5 years. The dose prescribed is 200 μg/kg, given as a single dose, which may be repeated after seven days. For immunosuppressed patients, two doses should be used at a one-week interval. It is the gold standard in the treatment of crusted scabies, in which it is associated with topical keratolytics, such as 5% salicylated petroleum jelly.

Ivermectin may also be used topically, at 1%, in propylene glycol or as a lotion. It should be applied on the entire body and the application should be repeated after one week.^14^

New medications are currently being investigated. The use of *Tinospora cordifolia* lotion showed similar efficacy to permethrin, with no side effects in treatment.^15^ Moxidectin is another promising medication whose efficacy and safety have already been demonstrated.^16^

Potent topical corticosteroids, two to three times daily, are used in the treatment of nodular scabies. Occlusion or triamcinolone infiltration (3–4 mg/mL) can also be used.^17^ Topical pimecrolimus and tacrolimus have been used in cases of corticosteroid resistance, with good results.^18^ In resistant cases, obeying the legal restrictions, oral thalidomide at a dose of 100 mg daily may be used.

## Pediculosis

Humans can be parasitized by three species of the Anoplura suborder: *Pediculus humanus capitis*, the etiological agent of scalp pediculosis, *Pediculus humanus,* which causes pediculosis corporis, and *Pthirus pubis*, the pubic louse. All feed on blood.

### Etiology and pathogenesis

#### Scalp pediculosis

**Figure 4:**
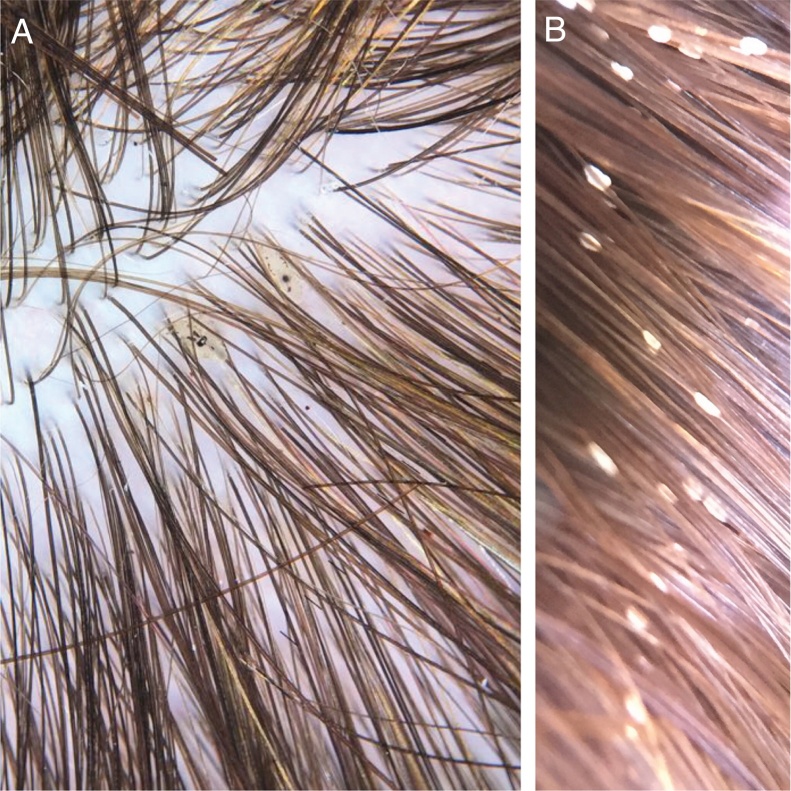
(A) *Pediculus capitis* on the scalp (Photo courtesy of Dr. Daniel França). (B) Nits, attached to the hair.

The etiological agent is *Pediculus capitis* (Fig. 4A). Louse saliva probably induces the pruritus that occurs on the scalp.

Nymphs and adults are difficult to see, but the eggs that attach to the hair are easily identified (Fig. 4B).

Wood's light and dermatoscopy can be used to aid diagnosis.

When a patient reports scalp pruritus, pediculosis should not be ruled out.^19^

#### Pediculosis corporis

By stinging the skin, *Pediculus humanus humanus* causes pruritus of varying intensity, leading to erythematous macules, papules, scabs, and abrasions, mainly seen on the trunk, armpits, and buttocks. Secondary infection, hyperpigmentation, and lichenification may occur. In Brazil, this condition is also termed the “homeless disease”.

The diagnosis is confirmed by finding the pedicle or nits in the folds of the clothes.

#### Pediculosis púbis or pthiriasis

The causative agent is *Pthirus pubis*, which parasitizes the hairs of the genitoanal region and eventually the hairs of the thighs, trunk, armpits, beard, eyelashes, eyebrows, and scalp borders.

The main clinical manifestation is pruritus. The diagnosis is made by observing the parasite in the skin, usually with the head portion inserted into the hair follicle, or nits stuck at the base of the hair.

In addition to abrasions, maculae ceruleae (bluish-gray spots) can be observed on the thighs and trunk, similarly to pediculosis corporis.

### Treatment

#### Scalp pediculosis

Topical pediculicides remain the main treatment.

Permethrin shampoo 1% should be left on the scalp for 10 min and then rinsed. Piperonyl butoxide 15% may also be used as shampoo.

Permethrin 5% may also be used, applied to the scalp at night, and removed the next day. The treatment should be repeated after seven to ten days, because during this period, the nits hatch. Malathion (0.5%) is an organophosphate widely used in the United Kingdom.

Due to the increasing resistance to pyrethroids, new products have been developed for the treatment of pediculosis.

Spinosad is an insecticide composed of the natural mixture of tetracyclic macrolides, spinosyn A and D. It interferes with nicotinic acetylcholine receptors, producing neuronal excitation and consequent lice paralysis, due to neuromuscular fatigue after long periods of hyperexcitation. Spinosad kills permethrin-susceptible and -resistant lice populations. It is also ovicidal. Spinosad is used at a concentration of 0.9% in suspension. It was approved by the FDA in 2011 for children aged 4 years or older. No systemic absorption was detected. It should be applied for 10 min, then rinsed. Treatment should be repeated after seven days.^20^, ^21^

5% benzyl alcohol in mineral oil was the first non-neurotoxic product approved by the FDA. It apparently acts by preventing the louse from closing its breathing spiracles, which allows the vehicle to penetrate and obstruct them, suffocating the parasites. As this is not an ovicidal, two 10-min applications should be performed, with a seven-day interval. It is FDA approved (Category B) and can be used from 6 months of age onwards.^22^

Another product, dimethicone (4% lotion), was approved in 2006; it was later marketed as a liquid gel. Its mode of action is still unclear. It may obstruct the respiratory spiracles, and the lice would thus die of asphyxiation. Another hypothesis is that it inhibits water excretion, causing physiological stress and death by paralysis or rupture of internal organs.^23^ The mode of application according to the according to the presentation: the gel should be applied for ten to 15 min; the lotion, for 8 h. The application should be repeated in seven to ten days. It has ovicidal action.^24^, ^25^, ^26^

Ivermectin topical 0.5% lotion was approved by the FDA in 2012 for the treatment of children aged 6 months or older. It should be applied for 10 min, and rinsed immediately after. It has ovicidal action.^27^

The use of a shampoo based on mineral oil and saponified olive oil was also effective.^28^

Single-dose ivermectin 200 μg/kg was also effective. It should be repeated after seven to ten days.

A recent study advocated the use of levamisole as a pediculicide.^29^

The nits must be removed. To facilitate the removal of nits, a vinegar solution at 50% can be used as well as a formic acid solution at 8%.

Brushes and combs should be placed in contact with pediculicides for ten to 15 min, and then washed with hot water.

#### Pediculosis corporis

Improved hygiene and washing the clothes promote healing.

#### Pediculosis or pthiriasis

It can be treated with permethrin 5% or deltamethrin 0.02% cream, applied at night and removed the following day. It is recommended to be used for two consecutive days, repeating after seven to ten days. Sexual partners should also be treated. The habit of shaving/waxing this area has led to a decrease in the number of cases.

In case of eyelash lesions, petroleum jelly can be used twice a day for eight days, mechanically removing the nits.

## Flea-induced dermatosis (pulicosis)

The bites cause hive papules in non-sensitized individuals. When sensitization occurs, especially in children, the salivary antigen is capable of causing local and distant lesions, causing acute infant pruritus and Hebra's prurigo.^30^

Some species can transmit disease-causing bacteria, such as plague, cat-scratch disease, and bacillary angiomatosis.

Corticosteroid creams and, if necessary, oral antihistamines are indicated for the treatment of flea bites.^31^, ^32^

For prophylaxis, an insecticide should be applied to the dwellings, and pets should receive treatment to eliminate fleas.

### Tungiasis

**Figure 5:**
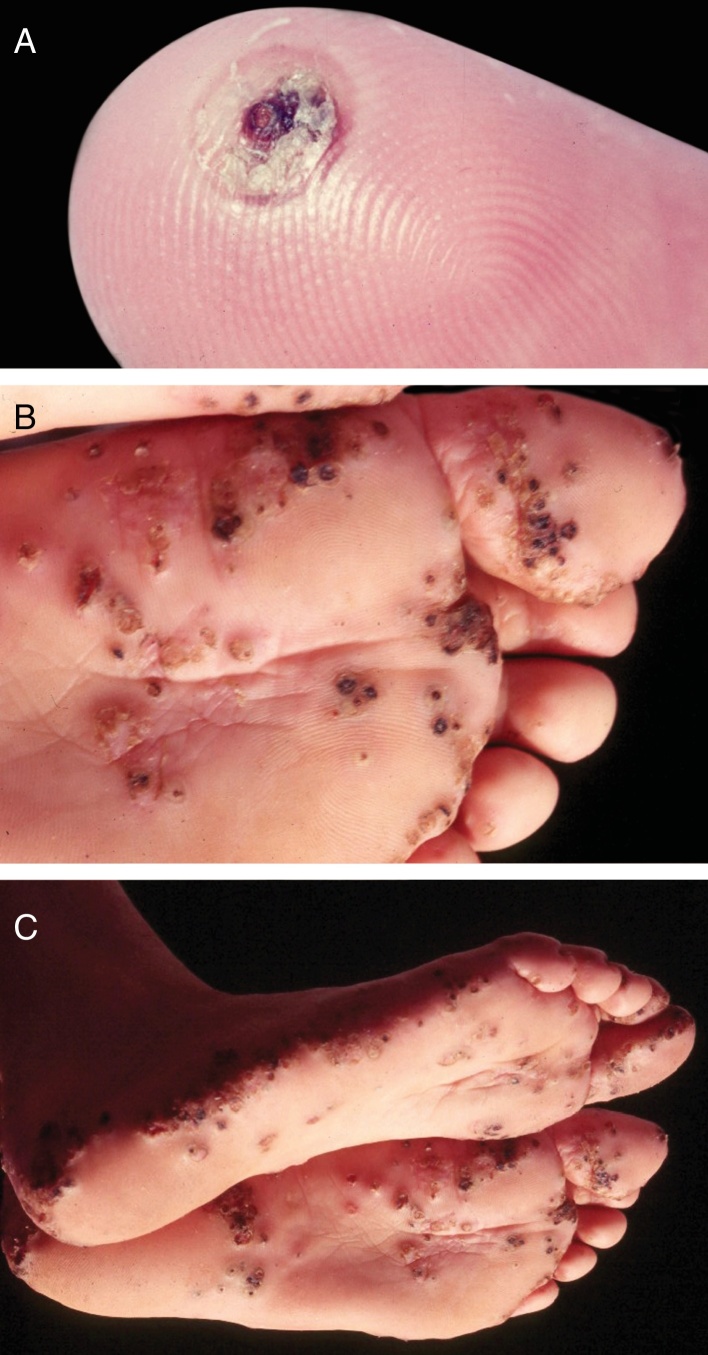
(A) Tungiasis – typical aspect. Isolated lesion. Note a pustule, in regression, with central crusted area. (B, C) Tungiasis. Multiple lesions, isolated and confluent.

It is caused by *Tunga penetrans*, the smallest of fleas; it measures on average 1 mm, and lives in dry and sandy places, especially in rural areas, in pig pens and corrals. The main hosts of these hematophagous fleas are pigs and humans. After feeding, the male leaves the host; the fertilized female penetrates the skin, introducing its head and chest into the epidermis, leaving out the respiratory stigmas and the egg-laying orifice.^33^ The eggs develop and the abdomen dilates, showing a yellowish nodule with a blackened spot in the center (Fig. 5A). There is pruritus and eventually pain. They are usually observed in the nail folds of the toes, interdigital spaces, and plantar regions.^34^, ^35^, ^36^ When many nearby lesions occur, they resemble a honeycomb (Fig. 5B and C). Secondary infections may occur, and the lesions serve as a gateway to other diseases.^37^ After the eggs are fully developed, the flea begins to expel them within two weeks; subsequently, the female dies.

### Treatment

The flea is removed with a needle, and an antiseptic is applied to the wound. In generalized cases, oral thiabendazole 25 mg/kg is used for ten days.^38^

Prophylaxis consists of wearing shoes.

Recent studies have shown that low viscosity dimethicone (NYDA) applied for seven days is effective and safe.^39^

### Bedbug dermatitis (cimicidiasis)

All cimicids (bed bugs) are blood-sucking parasites of birds and mammals. Two thirds of the species are bat parasites. The genus *Cimex*, with the species *lectularius and hemiptera*, is a human parasite. Commonly known as bed bugs, these insects have nocturnal habits and live in the cracks and holes of furniture and mattresses. At night, especially at dawn, they bite humans. During the meal they inject saliva, which contains an anticoagulant and anesthetic.

**Figure 6:**
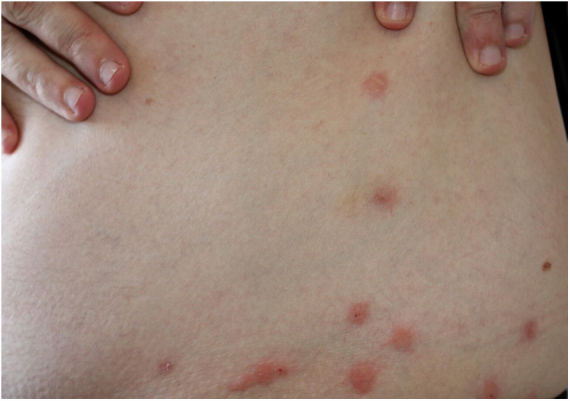
Bedbug dermatitis. Multiple, characteristic linear erythematous papular lesions located in the abdomen.

These bites are most commonly observed on the face, neck, arms, and hands. They cause hives and pruritic lesions (Fig. 6), often in linear arrangement. Distant sensitization injuries may occur, including bullous lesions.^40^, ^41^

Bed bugs share important traits with triatomine insects, but it is unclear whether these similarities include the ability to transmit *Trypanosoma cruzi*, which causes Chagas disease.

A recent study demonstrated efficient and bidirectional transmission of *T. cruzi* between hosts and bed bugs. Most bed bugs that fed on infected mice acquired the parasite; most mice were infected after cohabitation with exposed bed bugs. *T. cruzi* was also transmitted to mice after the feces of infected bedbugs were applied directly to the skin of the host. These findings suggest that bed bugs may be a *T.* *cruz*i vector.^42^

Corticosteroid cream is used for the treatment, and, depending on the pruritus, antihistamines may also be used. Bed bugs must be eradicated with the use of insecticides.

Several reports suggest an increase in the number of cases worldwide, including in Europe and the United States.^43^, ^44^

### Myiasis

Myiasis is characterized by the invasion of Diptera larvae into the skin, mucous membranes, and organs of humans and animals.

Among the various families of Diptera, the flies are noteworthy because, beside other diseases, they cause myiasis.

According to the evolutionary cycle of the Diptera flies, myiasis is classified into primary and secondary.

In patients with primary myiasis, the larvae invade healthy tissues. This variety is called furunculoid myiasis. In the secondary form, known as cavity myiasis, the flies lay their eggs on skin wounds or in the mucosa.^45^, ^46^

#### Furunculoid myiasis

**Figure 7:**
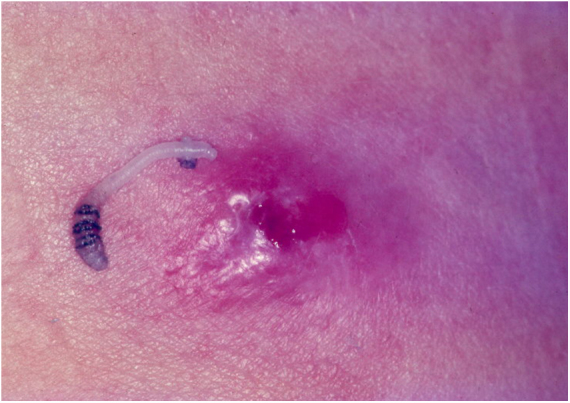
Furunculoid myiasis. Ulcero-nodular lesion and etiological agent (*Dermatobia hominis*).

It is observed in tropical regions of the American continent, extending from southern Mexico to northern Argentina. The life cycle of *D. hominis* is unique. After copulation, the female flies and captures a hematophagous Diptera fly. It lays 10–50 eggs in the prey's abdomen, without affecting its ability to fly.^47^ When it lands on the skin of a human or animal, the eggs hatch and larvae are released into the host through the hair follicles or the insect bite hole. Larva penetration is usually not noticed. At the larva entry site, an erythematous, papulous, pruritic, and painful lesion arises. The papule increases in size, evolving to a furunculoid aspect, with minor ulceration and outflow of serous exudate. In this opening, it is possible to observe the tail of the larva. The larvae feed on hypodermic material for an average of five to 12 weeks. After this period, the larvae leave the host and fall to the ground, becoming a pupa. Between 60 and 80 days, the pupa evolves into a winged insect. The larva can be exposed by pressing the lesion (Fig. 7).^47^, ^48^

The larva actively moves, and patients report a “stinging pain” at the site. Eventually, a secondary infection may occur, with abscess, cellulitis, and adenopathy.

When the larva leaves the nodule, the lesion regresses and heals.^47^, ^48^

The treatment consists of removing the larva, which can be done by compressing the nodule after a small incision into the lesion orifice. Hole obstruction with petroleum jelly, and placement of adhesive tape, preventing the larva from breathing, facilitates its removal. A lay treatment consists of placing heated bacon over the hole of the lesion – to breathe, the larva penetrates the bacon.

#### Secondary myiasis

It is caused by the larvae of flies that are not mandatory parasites. Depending on where the eggs are laid, these secondary infections can be cutaneous or cavitary. When eggs are accidentally ingested, intestinal myiasis may occur.

In the cutaneous form, the fly lays eggs on skin ulcerations. The eggs hatch and the larvae develop. The main etiological agents are the larvae of the flies *Cochliomya macellaria*, *C. hominivorax*, and other species of the family Sarcophagidae and genus *Lucilia*.^49^

The diagnosis is clinical, as the larvae are easily visualized. Traditionally, treatment is performed by removing the larvae after topical application of ether. The use of ivermectin 1% in propylene glycol is another method that has been used – 2 h after application, the lesion is cleaned and the larvae removed.^50^

#### Cavity myiasis

In these cases, the fly lays eggs in natural cavities, such as the nostrils, ear, eye sockets, and vagina. The most severe cases are caused by the species *C. hominivorax*.

The treatment of choice is a single dose of ivermectin 200 μg/kg.

Prior to ivermectin, mercury oxycyanide 1% was used.

## Demodicidosis

It is caused by *Demodex folliculorum*, a mite, which is a holoparasite of the hair follicle. These mites have a preference for areas with high sebum production, such as the face and chest.

*Demodex* has been frequently implicated in the etiopathogenesis of rosacea.^51^, ^52^, ^53^, ^54^ However, the *in vitro* resistance of the mite to high concentrations of metronidazole casts doubt on the role of the parasite in the pathogenesis of rosacea.^55^

It is possible that through the obstruction of the follicular ostia, *Demodex* contributes to the inflammatory reaction observed in rosacea, allowing bacterial proliferation or inducing mechanisms of hypersensitivity to mite antigens.

*D. folliculorum* has been attributed a pathogenic role in pityriasis folliculorum, a dermatological condition which is predominantly observed in middle-aged women. This dermatosis is characterized by the presence of diffuse erythema and folliculitis on the face. Microscopic examination evidences a large number of mites. Topical acaricides are used for treatment.^51^

Papular or papule-pustular eruptions on the face, trunk, and limbs observed in immunosuppressed individuals (HIV-positive patients, children with leukemia, and a case of mycosis fungoides) have also been attributed to *Demodex*.^56^, ^57^

Systemic treatment with ivermectin (200 μg/kg bodyweight, single dose) or metronidazole 250 mg daily, with varying duration, depending on the response, is effective.

## Cutaneous larva migrans

It is also called serpiginous linear dermatitis, creeping eruption, ground itch, sandworm, and plumber's itch.

Infection occurs when individuals come into contact with sand or soil contaminated with dog and cat feces.

During the rainy season, the number of infected people increases, probably due to the dissolution of dog and cat feces, facilitating the hatching of eggs and the penetration of larvae into people's skin.

The disease occurs by skin penetration of larval forms of dog and cat nematodes that are likely to penetrate the skin, probably by hyaluronidase secretion.^58^

*Ancylostoma brasiliensis* is the most common etiological agent. *A. caninum*, *Uncinaria* (European dog worms), *Bunostomum* (cattle worm), and *Phebotumum stenocephala* may also cause the disease.

### Clinical manifestations

**Figure 8:**
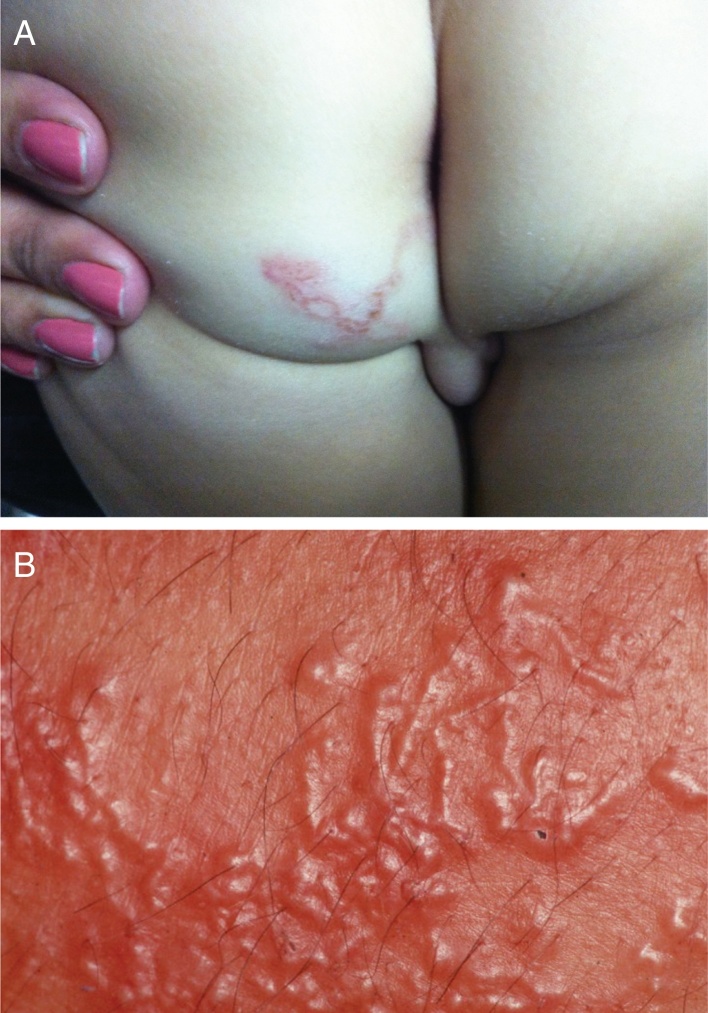
(A) Larva migrans. Intense pruritus. Typical serpiginous lesion with linear aspect. (B) Larva migrans. Numerous lesions caused by multiple larvae.

The lesions are usually linear, raised, erythematous, and serpiginous (Fig. 8A). Vesicles and even blisters may also appear. The most affected areas are the feet, legs, buttocks; it appears less often in other regions, such as the face, armpits, and penis. One case with oral mucosa lesions was described. Larvae dislocation triggers intense pruritus.^59^

When eczema and secondary infection occur, diagnosis can be very difficult. In such cases, it is first recommended to use ointments for the allergic condition, and antibiotics, if necessary. This will make the clinical aspect of larva migrans more evident.

When interdigital spaces are affected, maceration may occur. This clinical presentation may be confused with foot dermatophytosis.

Hematological alterations may also occur, with eosinophilia that, in some cases, reaches 30%. In severe infestations (Fig. 8B), the larvae may invade the blood stream and trigger Loeffler's syndrome, characterized by eosinophilic pneumonia associated with blood eosinophilia.^60^

### Treatment

Depending on the number of lesions and their location, treatment may be topical or systemic. In patients with multiple lesions or involvement of hyperkeratotic areas, such as the palmoplantar regions, systemic treatment is recommended.

Albendazole at a dose of 15 mg/kg/day for three days is a good therapeutic option. Its use does not prevent breastfeeding, and the fetal risk is category C. The cure rate varies from 77% to 100%.^61^, ^62^

Ivermectin, at a single dose of 200 μg/kg, is also effective. Depending on the evolutioncourse, the same dose may be repeated after seven days.^63^

When the number of lesions is reduced and located in the glabrous skin, topical treatment with thiabendazole 5% ointment may be indicated.

Carbonic snow or liquid nitrogen are also used.^64^, ^65^

## Lyme disease

Lyme disease (LD), also termed Lyme borreliosis, is a tick-borne zoonosis, mainly of the genus *Ixodes*, infected with spirochetes of the *Borrelia burgdorferi sensu lato* complex.^66^ Currently, 20 species have been recognized within the *sensu lato* complex, six related to the disease in humans: *B.burgdorferi* *strictu sensu* and *B.mayonii* (United States); *B.bavariensis*, *B. garinii*, *B.afzelli,* and *B.spielmanii* (Europe).^67^

Afzelius, in Sweden, in 1909, and Lipschutz, in Austria, in 1913, described the first cases of patients with centrifugal-growing erythematous plaques, which were termed chronic migratory erythema (CME).^68^, ^69^ In 1977, Steere et al. observed an association between CME and arthritis. The cases were studied in the city of Lyme, Connecticut (USA). Since this publication, the names Lyme arthritis and LD became popular. In addition to the association with arthritis, Steere et al. observed non-specific symptoms (malaise, fatigue, headache, fever, and other manifestations), as well as cardiac, ophthalmic, and neurological alterations.^70^ Due to the not always chronic evolution of skin lesions, in 1989, Detmar et al. proposed the name migratory erythema (ME), which has been widely adopted.^71^ In Brazil, the first cases were reported in Manaus by Talhari et al.^72^, ^73^ Filgueira, Azulay, and Florião^74^, ^75^, ^76^ also described clinically compatible cases in Rio de Janeiro. In 1992, the first cases of Brazilian patients with joint manifestations associated with *B. burgdorferi* infection were described.^74^

### Pathogenesis

The etiologic agent of ME/LD, a spirochete, was first isolated by Burgdorfer in 1982 in the intestine of *Ixodes dammini* ticks.^75^ This spirochete is now called *Borrelia burdorgeri sensu lato*. It has been identified in biopsies of skin lesions, blood, cerebrospinal fluid, synovial tissue, myocardium, and eyes of patients with LD.^12^

In the United States, mice and deer are important reservoirs of this spirochete. Elevated serological titers for *Borrelia* have been observed in horses, cows, sheep, and cats. In Brazil, wild rodents and other mammals, such as skunks, appear to participate in the epidemiological cycle of LD.^76^

The main transmitters of the disease are *Ixodes* ticks. In Europe, *Ixodes ricinus* is the most prevalent, while in the United States it is the *Ixodes dammini*, also known as *I. scapularis*. In Brazil, *Ambylomma cajannense* is the tick believed to be responsible for the transmission of LD. However, the participation of other tick species is not excluded. The evolutionary forms of ticks most associated with Borrelia transmission are nymphs and adult ticks. Nymph bites are painless, which would explain the fact that many infected patients do not remember being bitten by ticks.^77^

### Clinical picture

The cutaneous manifestations of LD are divided into early localized (erythema migrans and lymphocytoma cutis); early disseminated initial (ME and multiple lymphocytomas, which may be accompanied by alterations in other organs); and late (acrodermatitis chronica atrophicans).^78^

### Dermatological manifestations

**Figure 9:**
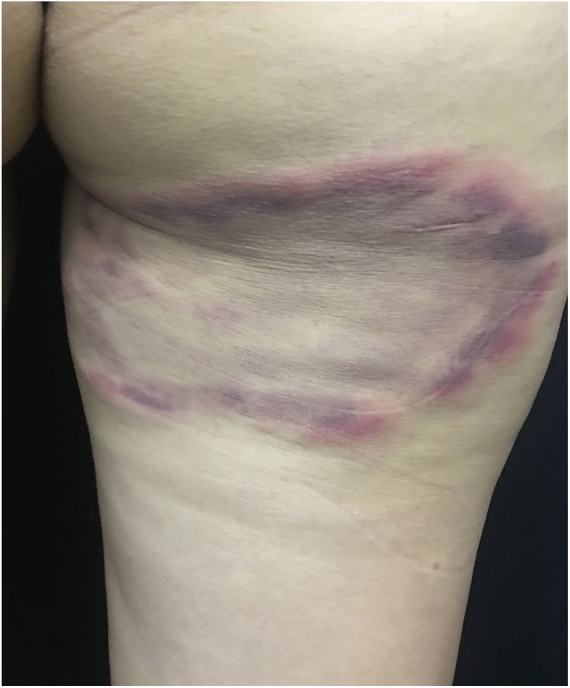
Lyme disease. Plaque presenting centrifugal growth, with erythematous-violet borders, measuring approximately 18 cm, located on the posterior surface of the thigh.

ME is the main early clinical manifestation of LD. Three to 30 days after the tick bite, an enlarged papule or small erythematous plaque appears at the site of inoculation, forming a plaque with discontinuous edges and a clear, cyanotic, and/or scaly center that expands centrifugally and may reach a large diameter (Fig. 9). Rapid progression of lesions, which may reach 20–30 cm or more in days or weeks, is common. In most patients, these lesions are asymptomatic. Different clinical features have been described in ME: erysipeloid, erythematous, lichenoid.^79^ European cases of ME tend to present with a small number of lesions and without a tendency to cutaneous spread.^80^

In addition to ME, another important cutaneous manifestation of the early phase of LD is lymphocytoma cutis, also called lymphadenosis benign cutis, which simulates B lymphocytic pseudolymphoma. Clinically, it is characterized by a single lump or erythematous plaque, 1 to 5 cm in diameter, usually located on the face, pinna, scrotum, or mammary areola. Lymphocytoma is often associated with infection by *B. afzelli* and *B. garinii*.^81^ In 2007, a case of cutaneous lymphocytoma in association with LD was published in Brazil.^82^

In this acute phase, systemic manifestations such as asthenia, arthralgia, myalgia, skin rash, adenopathy, splenomegaly and signs of meningeal irritation may also be observed. Early LD lesions may disappear without treatment, and manifestations of the second and third stages may appear months or years after initial infection. The main alterations include articular, cardiac, neurological, ophthalmological, and skin involvement. More rarely, late changes may occur in the presence of ME lesions.^83^ Among the manifestations of the late, cutaneous phases, acrodermatitis chronica atrophicans (ACA), also known as Pick-Herxheimer disease, is more associated with *B. afzelii* infection and is generally described in Europe. ACA is more common in adults and may manifest from six months to eight years after the tick bite. Clinically, it initiates with an erythematous plaque, evolving with skin atrophy and very prominent blood vessels, particularly in the lower limbs. The face and trunk may also be affected.^84^

### Other dermatological diseases associated with Borrelia infection

B. burgdorferi infection has been associated with other dermatological diseases such as plaque scleroderma, lichen sclerosus, atrophoderma of Pasini and Pierini, cutaneous B-cell lymphoma, and granuloma annulare.^85^ In a study conducted in Manaus in 2009, patients with scleroderma and atrophoderma of Pasini and Pierini were analyzed by immunohistochemistry with anti-*B. burgdorferi* polyclonal antibody; the presence of spirochete was confirmed in samples from both diseases.^86^

### Diagnosis

The diagnosis of the disease is based on epidemiological, clinical, and laboratory aspects. Laboratory diagnosis is based on serological tests (detection of specific antibodies) and/or on the finding of the etiological agent. In addition to serology, it is important to conduct histopathological and immunohistochemical tests, culture, and, if available, PCR.^87^

Detection of IgM or IgG anti-*B. burgdorferi* antibodies is commonly used for serological diagnosis and epidemiological investigation. Enzyme-linked immunosorbent assay (ELISA) tests are most commonly used; however, they present false-positive results in view of cross-reactions with other diseases, such as collagenoses, leishmaniasis, and syphilis. Thus, in non-endemic areas, for the definitive diagnosis it is necessary to perform a confirmatory exam that demonstrates the presence of the agent.^88^

Histopathological examination of ME lesions reveals proliferation and dilation of blood vessels associated with a central inflammatory infiltrate consisting of macrophages, mast cells, neutrophils, plasma cells, lymphocytes, and rare eosinophils. A pPCRymphocytic vasculitis may also be observed. In older lesions, atrophy of the epidermis and dermis may occur, as well as decreased dermal inflammatory infiltrate.^89^

PCR has been used to detect *Borrelia* nucleic acid sequences, with high specificity. However, the sensitivity of this diagnostic method is variable (20–81%). In 2008, Cerar et al. demonstrated that nested-PCR, using the flagellin gene, presented higher sensitivity than PCR (64.6% *vs*. 24%). PCR positivity is higher when fragments of cutaneous or synovial membrane lesions are used. It is less sensitive when performed on paraffin blocks, blood, synovial fluid, or cerebrospinal fluid.^90^

The culture using Barbour, Stroenery, Kelly (BSK) medium or variations thereof presents 100% specificity, but with a relatively low sensitivity. Given the difficulties of the technique and the contamination of the material, the results are positive in approximately 45% of the cases.^91^

In 2007, through a specific immunohistochemistry test for the detection of *Borrelia* sp., associated with the floating focus microscopy (FFM) technique, Eisendle et al. obtained results superior to nested-PCR in the identification of *Borrelia* (96% *vs.* 45.2%), with similar specificity (99.4% *vs*. 100%). The FFM consists of examining the slide in several planes simultaneously: horizontal and vertical, advancing and retracting the objective of the microscope, with magnifications of up to 400× under bright illumination. According to the authors, these simultaneous movements facilitate the detection of *Borrelia*.^92^ In 2010, using this same technique, Talhari et al. demonstrated, for the first time in Brazil, the presence of Borrelia in ME patients from Manaus, using immunohistochemistry with anti-*Borrelia* polyclonal antibody, and observation by FFM.^93^

### Treatment

The treatment of this disease is made according to the stage and clinical manifestation presented. In adult patients with localized LD, including ME cases without specific neurological manifestations, the recommended treatment is doxycycline (100 mg, 2× daily), amoxicillin (500 mg, 3× daily), or cefuroxime axetil (500 mg, 2× daily) for 14 days. For children and patients with doxycycline hypersensitivity, amoxicillin at a dose of 500 mg or 50 mg/kg/day, 3× daily is used; cefuroxime 500 mg or 30 mg/kg/day, 2× daily can also be used for the same period. Joint manifestations and cases of atrophic acrodermatitis are treated with the same antibiotics for 28 days. In cases of meningitis and other manifestations of early neurological LD, intravenous (IV) ceftriaxone 2 g/day or IV crystalline penicillin G at a dose of 18 to 24 million IU daily for 14 days is recommended. For the treatment of chronic erosive arthritis, sulfasalazine, chloroquine, methotrexate, and corticosteroids are recommended.^80^, ^94^

## Onchocerciasis

Onchocerciasis, also known as “river blindness,” is a chronic, non-contagious parasitic disease characterized by the presence of skin lesions, and often, severe ophthalmic lesions. It is caused by the filarial nematode *Onchocerca volvulus*.^95^

According to the World Health Organization (WHO), 198 million people are at risk of infection in 31 endemic countries.^96^ Onchocerciasis is the second leading cause of infectious blindness worldwide. Approximately 99% of *O. volvulus*-infected individuals live in sub-Saharan African countries, while the remaining patients live in Yemen, Sudan, and the Americas. In the latter, onchocerciasis has been endemic in six countries: Brazil, Colombia, Ecuador, Guatemala, Mexico, and Venezuela.^96^

Currently, the only onchocerciasis focus in the Americas recognized by WHO is the border region between Venezuela and the Brazilian states of Roraima and Amazonas, inhabited by members of the Yanomami tribe.^96^

Since 1974, the Onchocerciasis Control Program, developed by the WHO and funded by the World Bank and the United Nations, has promoted vector control and ivermectin treatment of onchocerciasis (used by the program since 1987) in 33 African countries.^95^ In 2016, over 133 million people living in risk areas received treatment for onchocerciasis.^97^

In 1990, the Onchocerciasis Elimination Program for the Americas, similar to the African model, was implemented to eliminate the disease in the thirteen endemic regions of the Americas.^96^ Since then, patients have been treated with ivermectin two or four times a year. In November 2017, the disease was considered eliminated in 11 of 13 areas with active transmission of *O. volvulus*.^96^

### Pathogenesis

Transmission of *O. volvulus* occurs through the bite of insects of the genus *Simulium.* These are found in greater abundance near the banks of river banks, hence the name “river blindness.”^95^ Microfilariae can be transmitted by different species of *Simulium*. In Brazil, the main species are *S. guyanense*, *S. incrustatum,* and *S. oyapockense*.^98^

Humans are the main hosts of the disease. By stinging an infected human, hematophagous females ingest microfilariae, which after two to three weeks become infective larvae. After a period of six to 12 months, these larvae develop into adult worms; males measure 2–4 cm in length and females, 40–50 cm. Adult worms, male and female, tend to lodge in interstitial spaces and adipose tissue, forming onchocercomas. They mate in these sites. After mating, females give rise to microfilariae, which migrate to connective tissue, superficial dermis, and the ocular globe. Each female can generate approximately one million microfilariae per year, which live up to 2.5 years. Adult females can live between nine and 16 years.^99^

### Clinical picture

**Figure 10:**
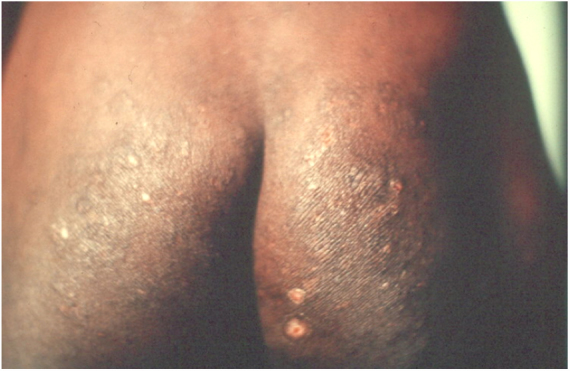
Onchocerciasis. Intense pruritus, presence of lichenification, exulcerations, and hyperpigmentation. Patient from the Infectious Disease Clinic, Ibadan, Nigeria (personal archive: Prof. Dr. Sinésio Talhari).

In the integument, the most common and early manifestation of onchocerciasis is chronic, constant pruritus that may simulate scabies (Fig. 10A). Areas with abrasions, lichenification, and hyperpigmentation are frequent. This condition is known as lizard skin.^100^, ^101^

**Figure 11:**
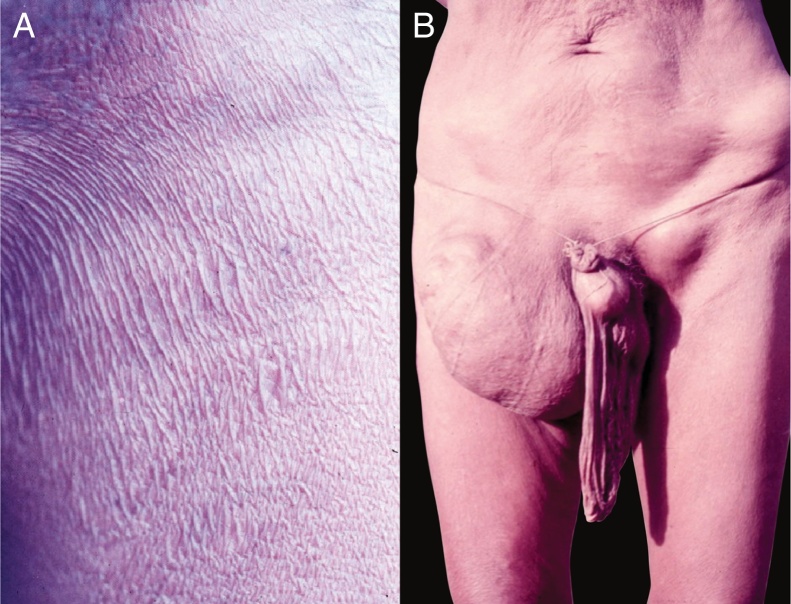
A. Onchocerciasis. Presence of atrophy, common in patients with long course. Native Brazilian from the Yanomami tribe (personal archive: Prof. Dr. Sinésio Talhari). B. Onchocerciasis. Observe the classic aspect of the “hanging groin” due to long evolution. There is also scrotum elongation (secondary to cutaneous atrophy), and there are nodules in the iliac crest and left groin – probably onchocercomas. Native Brazilian from the Yanomami tribe (personal archive: Prof. Dr. Sinésio Talhari).

Late depigmentation and acromy secondary to chronic pruritus are frequent, especially in the lower limbs (termed leopard skin). Atrophy may occur in the late stages of the disease, which may be mild (Fig. 10B) or very pronounced, leading to enlargement of the scrotum and inguinal hernia. Inguinal hernias are termed hanging groin (Fig. 11).^101^, ^102^ In some cases, lesions are limited to a certain skin area, especially on the leg, thigh, and gluteal region. This condition is known as sowda. Onchocercomas are painless, firm, rounded or elongated, and vary in size (0.5–10 cm in diameter). These lesions are usually located in the pelvis, lateral surfaces of the chest, and lower limbs in African patients; In the Americas, onchocercomas are mainly seen on the scalp, arms, and chest.^100^

The main ophthalmic lesions are keratitis, iridocyclitis, cataract, choroidoretinal lesions, post-neuritic optic atrophy, and amaurosis. Amaurosis produced by onchocerciasis is extremely common in Africa, resulting in great socioeconomic losses.^95^, ^101^

### Diagnosis

**Figure 12:**
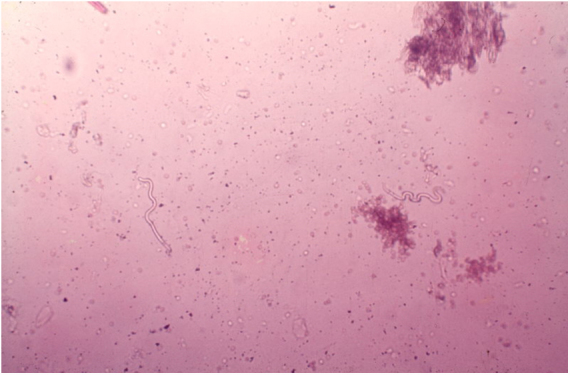
Onchocerciasis. Observe two microfilariae (Onchocerca volvulus). Direct examination in saline solution. 40× magnification (personal archive: Prof. Dr. Sinésio Talhari).

The diagnosis of onchocerciasis is based on the observation of microfilariae or the adult worm through direct examination. The procedure is simple and performed without anesthesia: the skin is pinched between the thumb and forefinger to prevent bleeding during material collection. A superficial fragment of skin is collected. The skin sample obtained is placed in saline solution and divided into small pieces with the aid of two scalpels. Microfilariae detection is done under the microscope without any staining at 40–50× magnification (Fig. 12).^101^ *O. volvulus* may also be seen by histopathological examination of the skin lesions and ophthalmic examination. The usual techniques of biopsy, 10% formalin fixation, and hematoxylin-eosin staining are employed.^101^, ^102^ The microfilariae can be identified by slit lamp examination.^101^, ^102^ Recently, immunological methods have been used to identify specific anti-onchocerca antibodies (ELISA and immunofluorescence) and onchocerca DNA (PCR).^103^

### Treatment

Treatment is primarily oral ivermectin, at a single dose of 150 μg/kg, every six months. This is a microfilaricidal drug and does not eliminate adult worms, which remain alive, producing new microfilariae.^95^ A randomized controlled trial suggests that the use of ivermectin every three months would eliminate more female adult parasites, further reducing *O. volvulus* transmission.^104^

## Financial support

None declared.

## Authors’ contributions

Alberto Eduardo Cox Cardoso: Approval of the final version of the manuscript; conception and planning of the study; elaboration and writing of the manuscript; critical review of the literature; critical review of the manuscript.

Alberto Eduardo Oiticica Cardoso: Approval of the final version of the manuscript; conception and planning of the study; critical review of the manuscript; critical review of the manuscript.

Carolina Talhari: Approval of the final version of the manuscript; conception and planning of the study; elaboration and writing of the manuscript; critical review of the literature; critical review of the manuscript.

Monica Santos: Approval of the final version of the manuscript; conception and planning of the study; elaboration and writing of the manuscript; critical review of the literature; critical review of the manuscript.

## Conflicts of interest

None declared.

## CME Questions

**Table:** 

1. Ivermectin, when used in the systemic treatment of scabies, should be used at the following dose:

a) 100 mcg/kg, in single dose.

b) 200 mcg/kg, in a single dose.

c) 200 mcg/kg, for seven days.

d) 20 mcg/kg, for seven days.



2. Immunosuppressed individuals when infected with *S. scabiei var. hominis* tend to develop:

a) Nodular scabies.

b) Bullous scabies.

c) Scabby scabies.

d) Classical scabies.



3. In scalp pediculosis, the main symptom and clinical findings are:

a) Scalp abrasions and pruritus.

b) Pruritus and presence of eggs (nits) on the hair.

c) Pruritus and blood crusts on the scalp.

d) Pruritus and cervical adenopathy.



4. In furunculoid myiasis, the treatment consists of:

a) Ivermectin - 200 mcg/kg, orally, three times a week.

b) Albendazole - single dose of 400 mg.

c) Thiabendazole - 25 mg/kg, for five days.

d) Larvae removal.



5. Among the drugs below, which one is not used to treat cutaneous larva migrans?

a) Ivermectin - 200 mcg/kg.

b) Albendazole - 400 mg single dose or 15 mg/kg/day for three days when the patient weighs over 60 kg.

c) Azithromycin - 500 mg/day, for five days.

d) Thiabendazole - 25 mg/kg for five days.



6. In pubic pediculosis or phthiriasis, what is the main finding to confirm the diagnosis:

a) Itching in the genital region.

b) Adenopathy of the ganglia in the inguinal region.

c) Finding the parasite in the skin with the head inserted in the hair follicle or the nits adhered to the hair base.

d) Finding bluish gray spots in the genital region.



7. The evolutionary forms of tick most often associated with transmission of *Borrelia budorgeri* are:

a) Larvae and adult ticks.

b) Nymphs and adult ticks.

c) Eggs and nymphs.

d) Nymphs and larvae.



8. Regarding migratory erythema, indicate the correct statement:

a) It appears three to five days after tick bite.

b) Lesions progress slowly and may reach up to 30 cm in diameter.

c) In most patients, migratory erythema is asymptomatic.

d) Histopathological examination is typical and confirms the diagnosis.



9. Regarding the clinical manifestations of onchocerciasis, the expressions sowda and lizard skin refer, respectively, to:

a) Depigmentation and late acromy.

b) Depigmentation and lichenification.

c) Upper limb injuries and lichenification.

d) Limited limb injuries and lichenification.



10. Regarding the treatment of onchocerciasis with ivermective, indicate the correct statement:

a) The drug is microfilaricidal.

b) It should be administered weekly for 6 months.

c) It should be administered every six months for 3 years.

d) It eliminates adult worms.

Answers:

Actinic keratoses: review of clinical, dermatoscopic, and therapeutic aspects. An Bras Dermatol. 2019;94(6):637–657.

**Table:** 

1. b	3. d	5. a	7. b	9. a
2. c	4. b	6. c	8. b	10. c
